# Andrological Aspects of Exercise: Moderate Swimming Protects against Isoproterenol Induced Testis and Semen Abnormalities in Rats

**DOI:** 10.3390/antiox11030436

**Published:** 2022-02-22

**Authors:** Péter Osváth, Miklós Szűcs, Denise Börzsei, Renáta Szabó, Zelma Nadin Lesi, Zsolt Turcsán, Médea Veszelka, Judith Sebestyén, Béla Juhász, Dániel Priksz, Csaba Varga, Anikó Pósa

**Affiliations:** 1 Department of Physiology, Anatomy and Neuroscience, Faculty of Science and Informatics, University of Szeged, 6726 Szeged, Hungary; dr.peter.osvath@kenezy.unideb.hu (P.O.); denise@exbio.bio.u-szeged.hu (D.B.); szrenata@bio.u-szeged.hu (R.S.); lesnad@exbio.bio.u-szeged.hu (Z.N.L.); turzso@exbio.bio.u-szeged.hu (Z.T.); veszmed@bio.u-szeged.hu (M.V.); vacs@bio.u-szeged.hu (C.V.); 2 Department of Urology and Andrology, Kenezy Gyula Campus, University of Debrecen, 4031 Debrecen, Hungary; dr.szucs.miklos@kenezy.unideb.hu; 3 HR-Pharma Ltd., 6726 Szeged, Hungary; 4 National Institute for Infectology and Haematology, Department of Burns and Plastic Surgery, South-Pest Hospital Centre, 1097 Budapest, Hungary; sebjud@exbio.bio.u-szeged.hu; 5 Department of Pharmacology and Pharmacotherapy, Faculty of Medicine, University of Debrecen, 4032 Debrecen, Hungary; juhasz.bela@med.unideb.hu (B.J.); priksz.daniel@pharm.unideb.hu (D.P.)

**Keywords:** swimming, infertility, inflammation, antioxidants, isoproterenol

## Abstract

The development and progression of male infertility are closely linked to a sedentary lifestyle; however, its underlying mechanisms are not fully elucidated. Our aim was to assess the protective effects of moderate swimming exercise on the male reproductive system in isoproterenol-treated rats. Male Wistar rats were divided into five groups as follows: (1) non-interventional controls (CTRL), (2) isoproterenol-treated (ISO), (3) pre-treatment swimming training + ISO (PRE + ISO), (4) ISO + post-treatment swimming training (ISO+POST), (5) pre-treatment swimming training + ISO + post-treatment swimming training (PRE + ISO + POST) groups. Testicular oxidative stress was induced by ISO injection (1.0 mg/kg). Rats in the pre- or post-training groups were trained five days a week. At the end of the experimental period, serum testosterone levels, sperms’ hyaluronan binding, and total glutathione (GSH) content, as well as myeloperoxidase activity (MPO), TNF alpha and IL6 concentrations in the testis and semen, were measured. Serum testosterone levels, sperms’ hyaluronan binding, and GSH content were found to be significantly reduced, while MPO, TNF alpha and IL6 concentrations in the testis and semen were elevated after the ISO treatment compared to the CTRL group. Moderate-intensity swimming exercise effectively alleviated the negative effects of high oxidative stress. Our findings provide the first evidence that moderate-intensity swimming exercise confers sustained protection from isoproterenol-induced adverse effects on testicular inflammation.

## 1. Introduction

Complex health benefits of physical exercise are well-established. Moderate exercise training decreases the risks of cardiovascular disorders and contributes to the prevention of non-communicable chronic diseases [1]. Previous clinical and experimental studies support the fact that a sedentary lifestyle plays a significant role in the rapid increase in the rate of infertility [2]. Epidemiological studies support that males are responsible for 20–30% of infertility cases and contribute up to 50% of cases overall [3]. A growing body of evidence suggests that a sedentary lifestyle has a detrimental effect on reproductive status in males, contributing to late-onset male hypogonadism, which is caused by diminished testosterone levels, poor libido, erectile dysfunction, and reduced sperm viability [4]. It has been documented, that physically active individuals have a higher percentage of motile spermatozoa compared to sedentary controls [5]. Moreover, moderate exercise training is shown to reduce DNA damage in sperms [6] and inflammation-related ageing [7] processes. Although fertilization is essential for the survival of all species, the underlying signaling pathways and biochemical mechanisms remain unclear.

In light of the sedentary lifestyle-related adverse mechanisms, there is a broad consensus that antioxidant/oxidant homeostasis, as well as inflammatory processes, are the main vulnerable targets and pathways that are affected by the unfavorable conditions. Our previous results prove that a sedentary lifestyle results in a disruption in oxidant/antioxidant homeostasis, which consequently causes oxidative damage [8,9]. As regards reproductive changes, oxidative stress can damage cell membranes via targeting membrane-involved polyunsaturated fatty acids. Testicular membranes are rich in polyunsaturated fatty acids; therefore, the testis is highly vulnerable to ROS-induced damage [10]. Among inflammatory parameters, tumor necrosis factor alpha (TNF-α) and interleukin-6 (IL-6) are key pro-inflammatory cytokines, which can be associated with male fertility. Azenabor et al. showed that in male gonads, cytokines are produced physiologically; however, adverse impacts can shift their concentration to the pathological level. Besides testicular expression, these changes can be observed in the semen, as well. 

In this study, a nonselective β-adrenergic receptor agonist, isoproterenol (ISO), was used to induce myocardial damage via transient ischemic events [8,11,12]. Our group previously underpinned that the underlying mechanisms of tissue damage are due to the imbalance of oxidant/antioxidant homeostasis and the accelerated inflammatory processes [13]. Furthermore, the ISO-induced abnormalities were successfully restored by voluntary physical exercise in female rats with estrogen hormone deficiency [8]. Besides the modulation of estrogen hormone-mediated processes, loss of endogenous testosterone level can be associated with reduced antioxidant capacity and elevated levels of inflammatory parameters [14]. Additionally, Cheng et al. found that ISO administration contributes to decreased testosterone levels and detrimental morphological changes in the testes [15]. Although steroid hormone (i.e., estrogen and testosterone) deficiency can be closely associated with shifts from antioxidant defense capacity to the production of reactive oxygen species, it is unclear whether regular physical exercise can contribute to inflammatory and antioxidant changes in the testicular tissue and the semen. Therefore, the current study was designed to investigate the hypothesized beneficial effects of moderate-intensity physical exercise (swimming) on testicular antioxidant status and inflammatory parameters, as well as on sperms’ vitality and testosterone concentrations.

Based on these findings, the same rat model and the dose of ISO was administrated to underpin the hypothesized beneficial effects of moderate-intensity swimming exercise on male fertility, as well as testicular and semen inflammatory parameters after ISO treatment. Our findings can serve as the first evidence that swimming exercise confers sustained protection from ISO-induced adverse effects on inflammatory parameters in the male reproductive system.

## 2. Materials and Methods

### 2.1. Animals and Exercise Protocol

All the experiments were reviewed and approved by the Animal Welfare Committee of the University of Szeged (XX./1405/2021). In this study, 8-month-old male Harlan-Wistar rats were used. Animals were kept at a standard 20–23 °C temperature, an appropriate light/dark cycle was provided, and husbandry conditions were accomplished according to Directive 2010/63/EU.

In our study, a maximum of 9 rats per group (the total number of the animals *n* = 45) were used. At the beginning of the study, the rats were divided into five groups as follows: (1) non- interventional controls (CTRL), (2) isoproterenol-treated (ISO), (3) pre-treatment swimming training + ISO (PRE + ISO), (4) ISO + post-treatment swimming training (ISO+POST), and (5) pre-treatment swimming training + ISO + post-treatment swimming training (PRE + ISO + POST) groups. Testicular morphological changes were induced by ISO injection; 1.0 mg/kg ISO (Sigma Chemicals Co., Poole, UK) diluted in 1 mL of physiological saline was injected subcutaneously. We have set the appropriate ISO dose in our previous studies, and prior to this current study, we have also verified in preliminary experiments that the dose used is not high but sufficient enough to induce myocardial damage [7]. During the study, all efforts were made to minimize the stress factors for the animals.

The animals were adapted to water one week before starting swimming training. The rats were then trained 5 days a week for 3 weeks individually, in a 20 cm × 20 cm pool filled with ~60 cm depth of water of 32–33 °C. The parameters of the pool and conditions used for swimming training were also determined and prepared on the basis of a previously accepted publication [15]. Each swimming training session lasted for 25 min, and all training sessions were performed in the mornings. After swimming, rats were dried with towels and placed back into their cages. Animals in the PRE+ISO group were trained for 3 weeks before ISO administration, while those in the ISO+POST group were trained for 3 weeks after the ISO injection, followed by a resting period. Rats in the PRE + ISO + POST group were trained for 3 weeks before and after ISO treatment, respectively. At the end of the total experimental period, semen samples were collected.

### 2.2. Measurement of Testosterone Level

At the end of the experimental periods, blood samples were collected in plastic serum separator gel blood collection tubes, centrifuged at 1000× *g*, 4 °C for 10 min. Then, total testosterone levels were measured with the Immulite 2000XPi (Siemens, Munich, Germany) chemiluminescent immunoassay and expressed as ng/dL.

### 2.3. Semen Collection

Artificial ejaculation was reached with electroejaculation, with a standardised method. The rats were placed on an electroejaculator platform in dorsal recumbency. The electroejaculator was equipped with a probe of 0.2 mm in diameter and 40 mm in length. The probe was lubricated with Vaseline and was inserted properly into the rectum. The electroejaculator’s output was set to 2–3 V. The semen was collected with a sterile pipette and was transferred to a 500 µL microfuge tube. Between 7 and 10 µL of the semen was used for the hyaluronan binding assay immediately, and the remaining quantity was stored at −20 °C for further biochemical analyses. After semen collection, the rats were placed back into their cages and were monitored daily. No adverse events occurred.

For our biochemical measurements and the hyaluronan binding assay, whole semen samples were used. 

### 2.4. Hyaluronan Binding Assay

A functional assessment of the semen was performed with the commercially available HBA^®^ tests (Biocoat Inc., Horsham, PA, USA). HBA assay is a suitable method to predict infertility. A low HBA score suggests a low proportion of mature sperm, while a higher HBA score shows the normal presence of healthy, mature sperm. The principle of this method is that in healthy fertile individuals, mature sperm bind to hyaluronan (the main component of the specific cells around the oocyte), while immature sperm do not bind. The hyaluronan-coated slide is designed to mimic the hyaluronan found around the oocyte; thus, under microscope bound sperm can be easily differentiated from unbound sperm. The test was carried out according to the manufacturer’s instructions. Briefly, 7–10 μL of semen was pipetted onto the hyaluronic acid-coated surface of the chambers. After 10–15 min of incubation at 24 °C, the binding rate of the sperms was determined under a microscope (Zeiss Imager Z.2 fluorescent microscope equipped with an Axiocam 506 mono camera).

The percentage of sperm binding to the hyaluronan layer is calculated as follows:

$$\%\;\mathrm{bound}\;=100\times \frac{\mathrm{bound}\;\mathrm{motile}\;\mathrm{sperms}}{\mathrm{bound}\;\mathrm{motile}\;\mathrm{sperms}\;+\;\mathrm{unbound}\;\mathrm{motile}\;\mathrm{sperms}}$$

### 2.5. Measurement of TNF-α and IL-6 Pro-Inflammatory Cytokine Concentrations

At the end of each individual period, the rats’ testicular tissues were excised after cervical dislocation. The excised tissue was homogenized in phosphate-buffered saline (PBS, pH 7.4), and the samples were centrifuged at 2500 rpm for 20 min at 4 °C. The supernatants and the semen were subjected to ELISA (GenAsia, Shanghai, China) and protein measurements. According to the manufacturer’s instructions, TNF-α and IL-6 concentrations were determined at 450 nm using an ELISA method. TNF-α and IL-6 levels were expressed as ng/mg protein content.

### 2.6. Measurement of GSH + GSSG Content

Total glutathione content (GSH+GSSG) indicating testicular antioxidant status was measured from the semen and testicular tissue samples. Testicular samples were homogenized in a solution of 0.25 M sucrose, 20 mM Tris, and 1 mM dithiothreitol (DTT) and were centrifuged at 15,000× *g* for 30 min at 4 °C. The supernatant fractions were collected and supplemented with 0.1 M CaCl_2_, 0.25 M sucrose, 20 mM Tris, and 1 mM DTT. After 30 min of incubation at 0 °C and further centrifugation at 21,450× *g* for 60 min at 4 °C, the clear fraction was used for the measurement of enzyme content. A solution containing 125 mM Na phosphate and 6.0 mM EDTA served as the diluent buffer for the stock solution of glutathione, glutathione reductase, 5,5′dithio-bis-2-nitrobenzoic acid (DTNB), and *β*-nicotinamide adenine dinucleotide phosphate (*β*-NADPH). A total volume of 40 μL per blank, standard, and semen/testicular samples and equal volumes of DTNB stock solution (20 μL) and *β*-NADPH (140 μL) were added to each well and the plate was incubated at 25 °C for 5 min. A total of 10 μL of glutathione reductase was used to start the reaction, and the absorbance was measured at 405 nm by a microplate reader 10 min after the initiation of the reaction. Total glutathione content was expressed as nmol/mg protein content. The measurement of GSH+GSSG content is based on a biochemical reaction (catalytic action) of GSH or GSSG in the reduction of DTNB by a mixture of glutathione reductase and NADPH [16].

### 2.7. Measurement of Myeloperoxidase Enzyme Activity

Rat testicular tissue samples were homogenized in ice-cold phosphate-buffered saline (PBS, pH 6.0), freeze-thawed three times, and then centrifuged twice at 15,000× *g* for 15 min at 4 °C. The supernatant fraction was discarded, and a 12 μL aliquot of the testicular supernatant or semen was added to a mixture of 280 μL of PBS and 0.167 mg/mL of O-dianisidine dihydrochloride. The reaction was started with 10 μL of 0.03% hydrogen peroxide, and the sample was assayed spectrophotometrically at 490 nm after 90 sec of shaking. Semen and testicular MPO activity were expressed as µU/mg protein content.

### 2.8. Protein Analysis

Using a commercial protein assay kit (Bio-Rad Labs, Hercules, CA, USA), aliquots (20 μL) of the diluted testicular and semen samples were mixed with 980 μL of distilled water and then, 200 μL Bradford reagent was added to each sample. After mixing and following a 10-min incubation time, the samples were assayed spectrophotometrically at 595 nm. The protein level was expressed as mg protein/mL.

### 2.9. Statistical Analysis

Statistics were performed using the GraphPad Prism software for Windows, version 7.01 (GraphPad Software Inc., La Jolla, CA, USA). All data are presented as the mean value of the group ± standard deviation (SD). The Shapiro–Wilk normality test was used to estimate Gaussian distribution. Analysis then was performed using a one-way analysis of variance (ANOVA) test followed by Tukey’s multiple comparisons post-test (when F achieved *p* < 0.05 and the normality test was passed). Statistical analyses were carried out only for experiments where each group size (n) was at least 5. Probability values (*p*) less than 0.05 were considered significantly different, and exact p values are shown for each group in scatter dot plots.

## 3. Results

### 3.1. Changes in Serum Testosterone Levels

**Figure 1:**
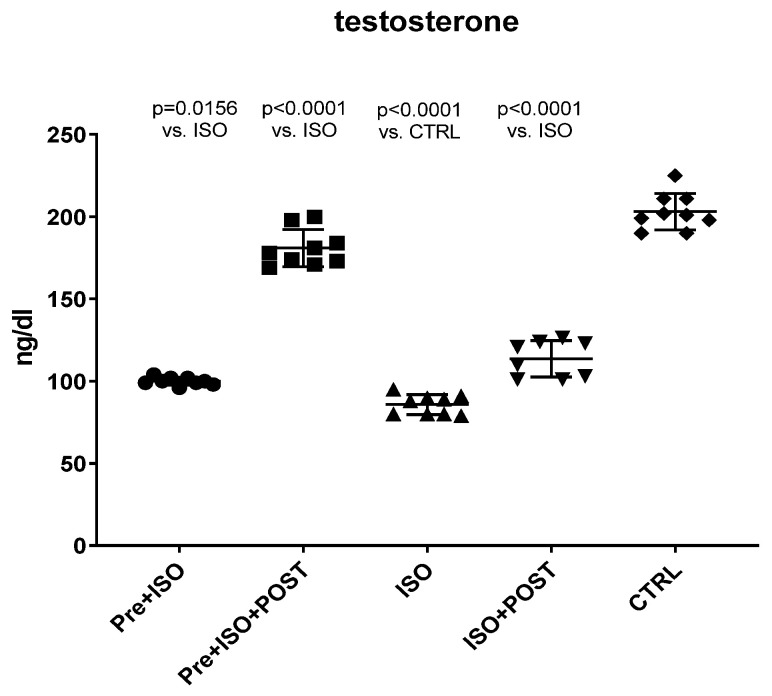
Changes in serum testosterone levels (ng/dL) as a result of isoproterenol treatment and exercise training. The results are shown as mean ± S.D., *n* = 8–9/group, one-way ANOVA, Tukey’s post-test. Significant differences between treatment groups are shown with exact *p* values. PRE = Pre-swimming, ISO = Isoproterenol injection, POST = Post-swimming, CTRL = Control group.

Serum testosterone levels were measured at the end of the experimental period in all groups. As shown in Figure 1, the serum concentrations of the main androgen hormone were significantly reduced as a result of ISO treatment compared to the CTRL group. Pre-treatment swimming or post-treatment swimming, separately, induced a significant increase in the level of testosterone compared to the non-trained ISO group. Moreover, the combination of pre- and post-treatment swimming resulted in twice as high androgen levels than either the pre- or post-treatment training.

### 3.2. Hyaluronan Binding Assay

**Figure 2:**
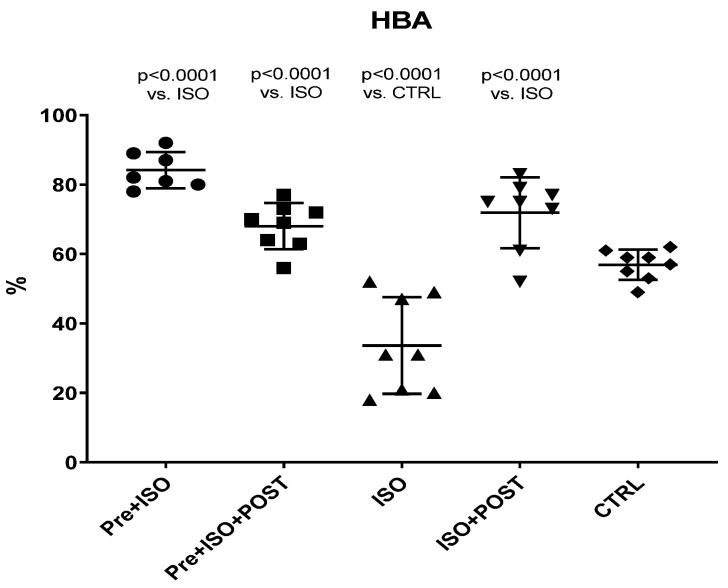
Changes (%) in the quality and maturity of the semen as a result of isoproterenol treatment and exercise training. The results are shown as mean ± S.D., *n* = 7–8/group, one-way ANOVA, Tukey’s post-test. Significant differences between treatment groups are shown with exact *p* values. HBA = Hyaluronan Binding Assay, PRE = Pre-swimming, ISO = Isoproterenol injection, POST = Post-swimming, CTRL = Control group.

Hyaluronan Binding Assay (HBA) scores were significantly decreased in ISO-treated animals compared to the CTRL group, while the 3-week-long swimming training, either before or after the ISO injection or in a combined manner, significantly increased HBA values (Figure 2).

### 3.3. Pro-Inflammatory Cytokine Assays

#### 3.3.1. Determining TNF-α and IL-6 Concentrations in the Semen

**Figure 3:**
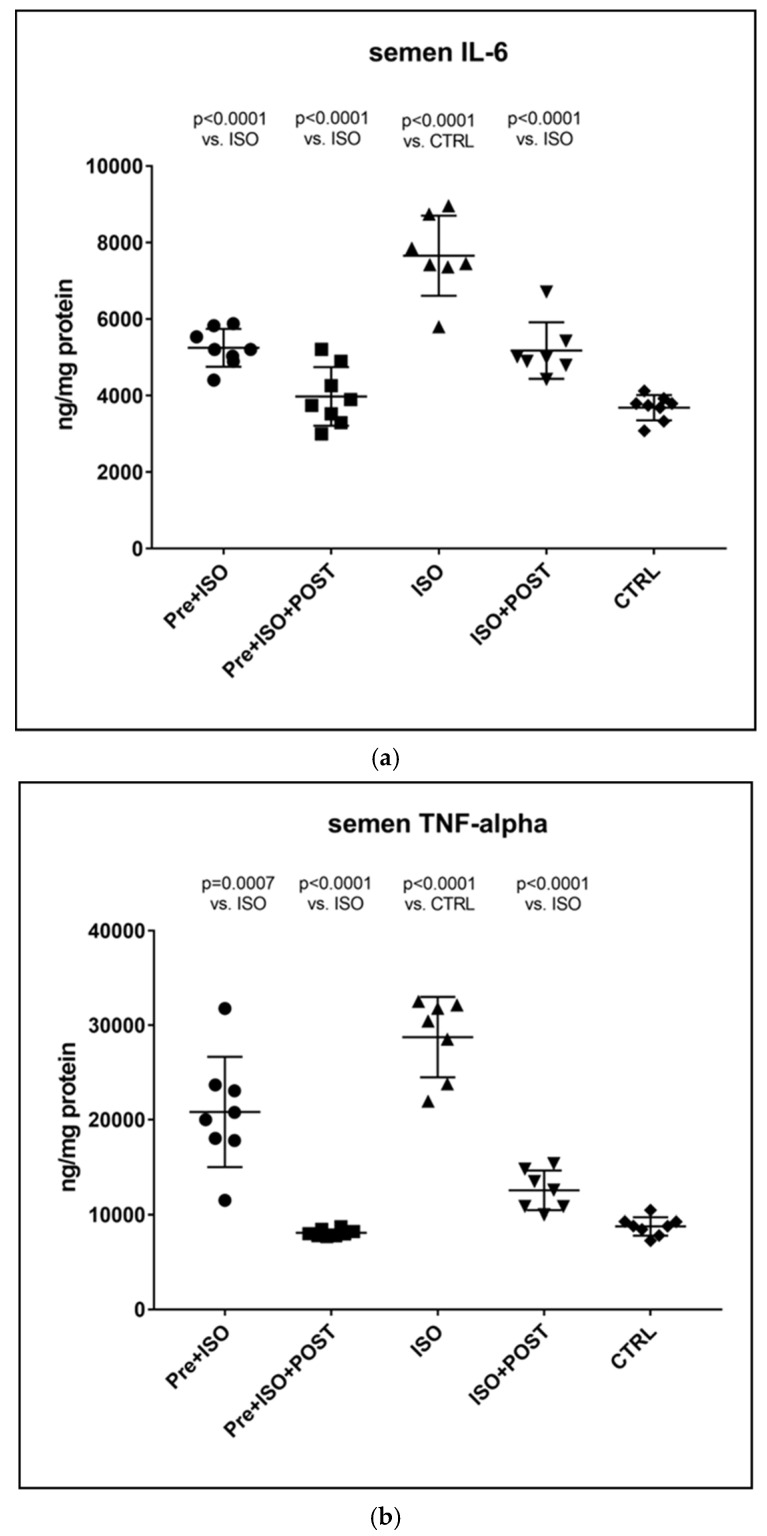
(**a**) The effects of isoproterenol treatment and exercise training on interleukin-6 (IL-6) concentrations of the semen, expressed as ng/mg protein. (**b**) The effects of isoproterenol treatment and exercise training on tumor necrosis factor-alpha (TNF-α) concentrations of the semen, expressed as ng/mg protein. The results are shown as mean ± S.D., *n* = 7–8/group, one-way ANOVA, Tukey’s post-test. Significant differences between treatment groups are shown with exact *p* values. PRE = Pre-swimming, ISO = Isoproterenol injection, POST = Post-swimming, CTRL = Control group, TNF-α = tumor necrosis factor-alpha, IL-6 = interleukin-6.

As expected, the TNF-α levels of semen specimens were significantly higher after ISO administration relative to the CTRL group, and similar results were obtained for the concentrations of IL-6, as well. However, three weeks of regular swimming exercise significantly mitigated these elevations of pro-inflammatory cytokines both in the PRE + ISO and POST + ISO groups. Combined pre- and post-treatment swimming training was found to exert the most considerable beneficial effects on TNF-α and IL-6 concentrations compared to the training-free ISO-treatment group. All the observed changes were found to be statistically significant compared to the ISO and CTRL groups (*p* < 0.0001 for both comparisons). Data are shown in Figure 3.

#### 3.3.2. Measurement of Testicular TNF-α and IL-6 Concentrations

**Figure 4:**
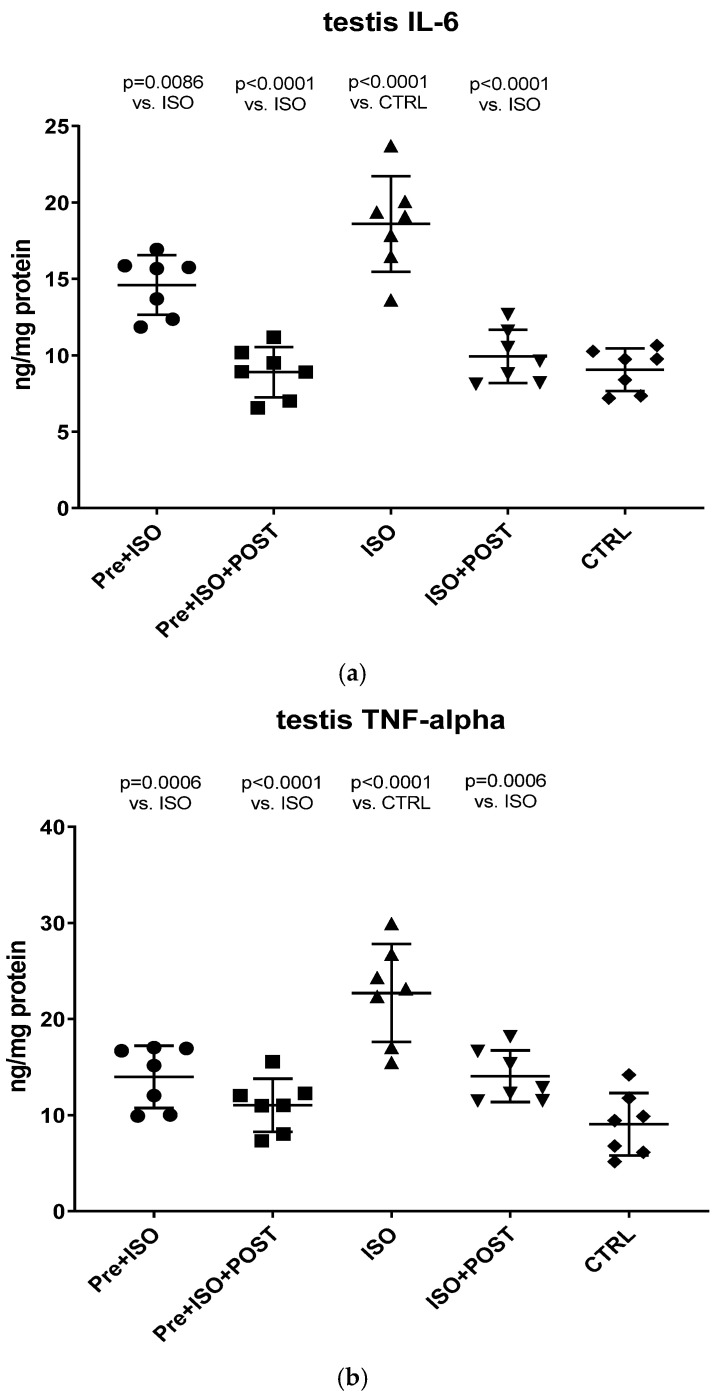
(**a**) The effects of isoproterenol treatment and exercise training on testicular interleukin-6 (IL-6) concentrations, expressed as ng/mg protein. (**b**) The effects of isoproterenol treatment and exercise training on testicular tumor necrosis factor-alpha (TNF-α) concentrations, expressed as ng/mg protein. The results are shown as mean ± S.D., *n* = 7/group, one-way ANOVA, Tukey’s post-test. Significant differences between treatment groups are shown with exact *p* values. PRE = Pre-swimming, ISO = Isoproterenol injection, POST = Post-swimming, CTRL = Control group, TNF-α = tumor necrosis factor-alpha, IL-6 = interleukin-6.

As shown in Figure 4a, a significant increase in IL-6 concentration within the testis was detected in the ISO-treated group compared to CTRL animals. Swimming exercise prior to or after ISO administration was found to mitigate this adverse effect of ISO, resulting in a significant decrease in testicular IL-6 concentrations compared to the ISO and CTRL groups. The combined training of pre- and post-treatment swimming was detected to be the most effective in terms of reducing ISO-induced elevated IL-6 concentrations (*p* < 0.0001 vs. ISO).

Similar to the changes of IL-6 concentrations, ISO treatment significantly increased TNF-α levels in the testis compared to the CTRL group (*p* < 0.0001). Again, swimming exercise training was found to be effective in terms of reducing TNF-α levels in the PRE + ISO and POST + ISO, and especially in the PRE + ISO + POST groups (Figure 4b).

### 3.4. Determination of MPO Activity

**Figure 5:**
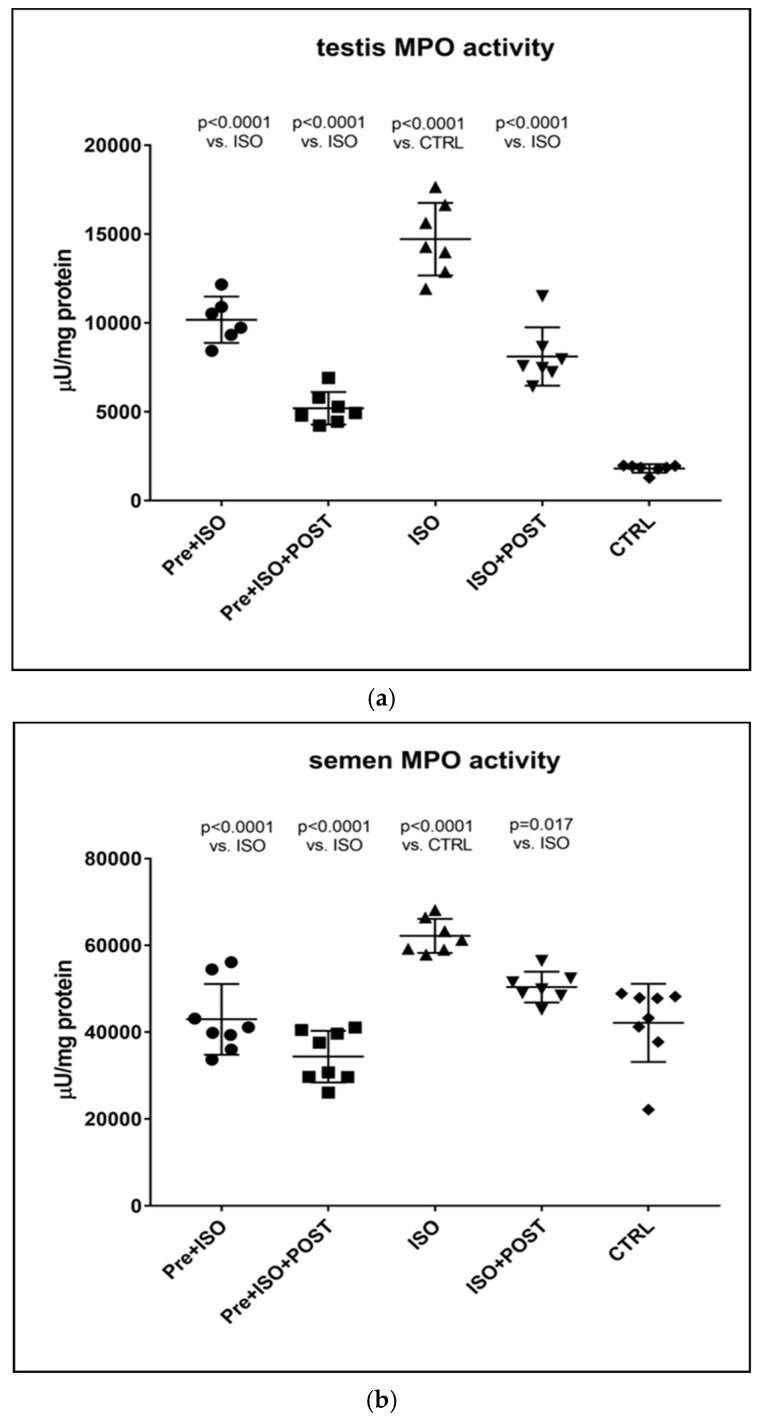
(**a**) The effects of isoproterenol treatment and exercise training on testicular myeloperoxidase (MPO) enzyme activity (expressed as: µU/mg protein). (**b**) The effects of isoproterenol treatment and exercise training on myeloperoxidase (MPO) enzyme activity of the semen, expressed as µU/mg protein. The results are shown as mean ± S.D., *n* = 6–8/group, one-way ANOVA, Tukey’s post-test. Significant differences between treatment groups are shown with exact *p* values. PRE = Pre-swimming, ISO = Isoproterenol injection, POST = Post-swimming, CTRL = Control group, MPO = myeloperoxidase enzyme.

Similar to the changes detected in inflammatory cytokine levels in the semen and testis, MPO activity was also significantly higher in ISO-treated sedentary animals compared to CTRL rats. Again, testicular and ejaculate-associated inflammatory processes were found to be mitigated by regular physical activity: a significant reduction in MPO activity was detected in the PRE + ISO and POST + ISO groups compared to the ISO and CTRL groups (*p* < 0.0001 for both comparisons). Furthermore, pre- and post-treatment swimming in combination elicited the largest decrease in MPO activity in comparison with the ISO group (*p* < 0.0001, Figure 5).

### 3.5. Evaluation of GSH + GSSG Content

**Figure 6:**
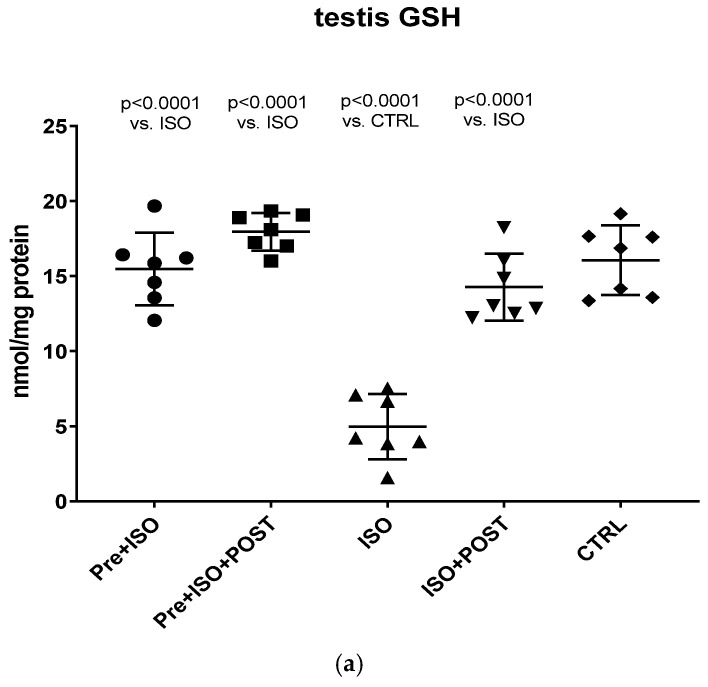

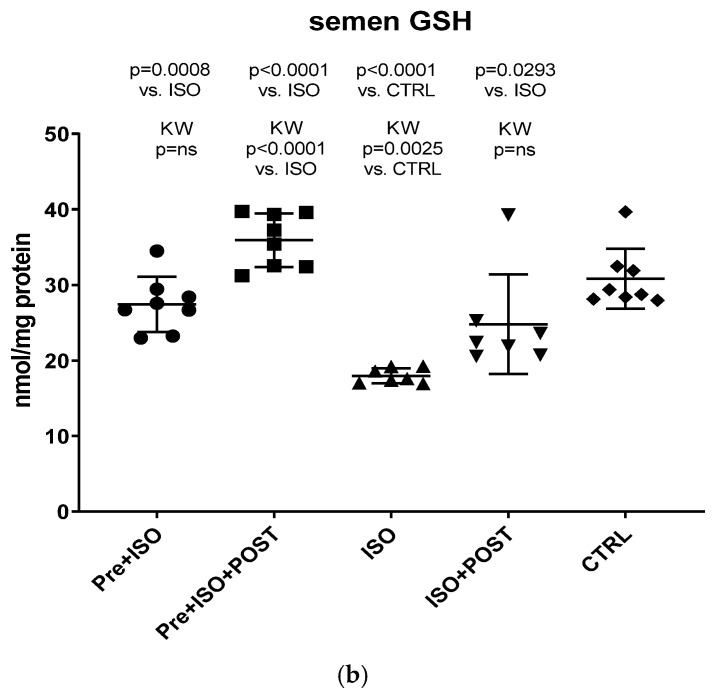
(**a**) The effects of isoproterenol treatment and exercise training on the testicular ratio of reduced/oxidized glutathione (GSH) content, expressed as nmol/mg protein. (**b**) The effects of isoproterenol treatment and exercise training on the ratio of reduced/oxidized glutathione (GSH) content of the semen, expressed as nmol/mg protein. The results are shown as mean ± S.D., *n* = 7–8/group, one-way ANOVA, Tukey’s post-test. Significant differences between treatment groups are shown with exact p values. PRE = Pre-swimming, ISO = Isoproterenol injection, POST = Post-swimming, CTRL = Control group, GSH = total glutathione.

Compared to CTRL animals, the level of GSH decreased significantly, both in the semen and testis (*p* < 0.0001); however, regular swimming training before or after ISO treatment induced a significant improvement in testicular and ejaculate antioxidant status. Again, the combined pre- and post-treatment training was found to result in the greatest improvement of GSH values: the changes were significant compared to both the CTRL and ISO groups (*p* < 0.0001 for both comparisons). Data are shown in Figure 6a for the testis and Figure 6b for the ejaculate.

## 4. Discussion

The present study demonstrates that ISO-induced oxidative stress promotes testicular inflammation and hormonal imbalance, characterized by low testosterone concentrations and an impaired antioxidant capacity within the testes, which eventually leads to a decrease in mature/immature sperm ratio. These detrimental changes can be associated with male infertility. Our findings regarding the negative effects of oxidative stress on the male reproductive system are in line with several studies indicating that oxidative stress causes testicular dysfunction. These studies also suggest that oxidative stress in the testicular milieu is associated with DNA damage and abnormal sperm production, accounting for the rising prevalence of reduced male fertility [16,17,18]. Moreover, oxidative stress factors are reported to play a causal role in the development of sperm abnormalities and abnormal function of the testes [19,20]. In a previous study, Wagner et al. summarized that ROS is a potential contributor to male infertility, which has been reported in the literature since the 1940s. Oxidative stress leading to defective sperm function was demonstrated in early studies illustrating the toxic effect of peroxyl (^•^ROO^−^) and hydroxyl (^•^OH) radicals, superoxide (^•^O_2_^−^) anion, H_2_O_2_, nitric oxide (NO), and peroxynitrite anion (ONOO^−^) on sperm metabolism and motility [21]. Irrespective of the causative agent, oxidative stress (e.g., ISO treatment in our experiment) disrupts the oxidant/antioxidant balance and influences the vascular tone and testicular blood flow. It is highly probable that decreased blood flow, as well as the direct oxidative damage of the testicular tissue, has an impact on testosterone levels. Specifically, decreased testosterone production should result from a direct negative effect of ISO on Leydig cells or from the reduction in blood supply to Leydig cells. It is also probable that ISO-induced oxidative stress and the consequently reduced blood flow to the testes, accompanied by a drop in testosterone secretion, decrease sperm hyaluronan binding, as demonstrated in our experiment. In addition, a significantly lower level of GSH was found in both the testis and the semen of ISO treated animals in our rat model, indicating that ISO-induced testicular damage destroyed the antioxidant protection of the tissue. This reduced testicular GSH level may also play a role in the impairment of testosterone biosynthesis after ISO treatment. GSH is a thiol-containing tripeptide present in virtually all cells, which has a crucial role in cellular antioxidant defense. As a key antioxidant, GSH removes hydrogen peroxide and organic peroxides from the cells.

Therefore, any decline in the level of GSH leads to an increased production of ROS [22]. In another experiment, it was also concluded that increased oxidative stress and the consequent antioxidant deficit might be a triggering factor in ISO-induced tissue damage [18]. Moreover, we have also demonstrated that ISO-induced oxidative stress generates inflammation via the upregulation of MPO, TNF-α, and IL-6, which should also contribute to the reduction in testosterone concentrations and the reduced ratio of mature sperm, leading to infertility. It should be noted that MPO is an enzyme expressed in neutrophil granulocytes and monocytes and is a key element of neutrophils’ oxidant production. Elevated serum levels of MPO are associated with inflammation, neutrophil infiltration, and increased oxidative stress [23,24]. Neutrophils activated by ROS, as seen in the ISO-treated group, exacerbate tissue injury via the production of oxygen metabolites and the activation of the cytotoxic MPO enzyme. In our study, increased MPO activity was detected after ISO treatment, suggesting that the oxidative stress caused by ISO in testicular tissues involves neutrophil accumulation.

However, in our rat model, the negative effects of ISO were found to be alleviated by moderate exercise training (swimming). Interestingly, literature data on the impact of exercise training on testicular function are controversial. It has been reported that high-intensity physical activity can contribute to the development of oxidative stress within the male reproductive system, thus it may interfere with male fertility [10]. Furthermore, intensive swimming training (3 h/day, 5 days a week for 6 weeks) was also demonstrated to induce oxidative stress in the male reproductive system [25]. In contrast, Yi et al. have reported that moderate-intensity exercise has the potential to alleviate testicular oxidative stress and inflammatory responses, while, again, high-intensity physical activity was found to be detrimental in this respect. Similarly, it has also been demonstrated that moderate-intensity exercise diminishes the negative effects of obesity on male reproductive function by decreasing testicular oxidative stress and inflammation [24].

In the present study, moderate pre- and/or post-treatment swimming was introduced to protect against ISO-induced augmenting inflammatory response and reduced capacity of the GSH-mediated antioxidant barrier. Notably, compared to other types of physical activities (e.g., running), swimming has the benefit of minimizing the accompanying testicular temperature rise, which is known to have a detrimental effect on sperms. To the best of our knowledge, our study is the first one aiming to clarify whether moderate swimming exercise has a protective role against oxidative stress-induced testicular inflammation in a rat model. The protective effects of physical activity on the cardiovascular system have been widely studied; however, its potential benefits on the male reproductive system are less well characterized. Our previous research focusing on the cardiovascular consequences of ISO-induced oxidative stress revealed that recreative running wheel exercise could mitigate the high ratio of myocardial infarct size [26] and ameliorate the antioxidant status after ISO treatment in ovary-intact and pharmacologically-induced estrogen-deficient female rats [8].

Our current findings demonstrate that either pre- or post-treatment swimming in itself is effective against ISO-induced adverse outcomes; however, the most significant benefit was evident in the combined PRE+POST swimming exercise group, indicating that moderate physical activity (swimming) has a strong potential to ameliorate ISO-induced inflammation and antioxidant capacity shift. Specifically, moderate swimming exercise was found to improve the antioxidant status by enhancing the reduced activity of the endogenous antioxidant enzyme GSH. Moreover, the increased MPO activity in the testes and semen was successfully reversed, at least to some extent, with moderate swimming exercise. To further support our hypothesis, serum concentrations of pro-inflammatory cytokines were also measured. The analysis of testicular tissues and semen in male rats revealed that moderate-intensity swimming exercise was associated with decreased levels of IL-6 and TNF-α, further confirming that regular physical activity has the potential to reduce testicular inflammation. These observations are consistent with the changes in GSH activity, serum testosterone levels, and the results of sperm HBA analysis. Our findings are in line with those described in a study by Yi et al. [27]. Previous in vitro studies have established that TNF-α is a potent inhibitor of Leydig cell function: it inhibits both P450scc and IGF-1 mRNA gene expression [28]. Based on these previous findings and the outcomes in this study, it is suggested that moderate swimming exercise can inhibit the pro-inflammatory cytokines by reducing oxidative stress, which can promote testosterone synthesis by enhancing anti-inflammatory cytokine. A study in males also showed that exercise training reduced the serum concentrations of neutrophils and decreased the synthesis of IL-6 and TNF-α [29]. Several studies point to IL-6 as a key player in this process, as it can modulate the production of other pleiotropic cytokines involved in the inflammatory process (e.g., TNF- α), which are secreted at the onset of the inflammatory response and act locally at the site of injury. In our study, we used ISO injection because the testicular tissues of infarcted male rats are in a state of high oxidative stress and high inflammatory response. It is supported by the elevated levels of MPO, IL-6, and TNF-α, as well as the decreased level of GSH. Besides the molecular and biochemical alterations of the testicular tissue, profound changes also appear in the semen. Recent studies support that a growing number of harmful environmental factors, such as heavy metals or air pollution, can further affect the nuclear basic protein of the semen, thus causing severe DNA damage and ultimately impairing sperm quality [30,31]. Besides environmental status, lifestyle is a detrimental factor in sperm maturation, morphology, and motility. We found that the percentage of mature spermatozoa significantly improved as a result of physical exercise.

The present study suggests that disruption of the antioxidant defense system and elevated inflammation have detrimental effects on the male reproductive system, which may manifest testicular damages and adverse changes in the semen. However, moderate swimming exercise training can have a significant protective effect against testicular oxidative stress and inflammation, as well as improve the mature/immature sperm ratio.

## 5. Conclusions

In conclusion, 1 mg/kg ISO injection down-regulated the production of the antioxidant GSH and up-regulated the MPO-IL6-TNF-α inflammatory pathway in the testes and semen, leading to stress-induced testicular damage. This is the first evidence to indicate that moderate-intensity physical activity (swimming) can effectively alleviate the negative effects of ISO-induced inflammation and antioxidant capacity damage on the male reproductive system, thus it may be utilized as a preventive and/or therapeutic strategy to counteract the adverse outcomes, such as oxidative tissue damage, inflammatory processes, reduced testosterone biosynthesis, and adverse mature/immature sperm ratio. Our findings give insight into the biochemical background of the beneficial effects of moderate swimming training, which may serve as an effective approach to improve male fertility.

## Data Availability

All data used to support the findings of this study are included within the article.

## References

[1] Lin Y.-Y., Lee S.-D. (2018). Cardiovascular Benefits of Exercise Training in Postmenopausal Hypertension. Int. J. Mol. Sci..

[2] Meikle A.W., Reis L.O., Gibson M., Peterson C.M., Carrell D.T., Hammoud A.O. (2012). Obesity and Male Infertility: A Practical Approach. Semin. Reprod. Med..

[3] Agarwal A., Mulgund A., Hamada A., Chyatte M.R. (2015). A unique view on male infertility around the globe. Reprod. Biol. Endocrinol..

[4] Hammiche F., Laven J.S., Twigt J.M., Boellaard W.P., Steegers E.A., Steegers-Theunissen R.P. (2012). Body mass index and central adiposity are associated with sperm quality in men of subfertile couples. Hum. Reprod..

[5] Vaamonde D., Da Silva-Grigoletto M.E., García-Manso J.M., Barrera N., Vaamonde-Lemos R. (2012). Physically active men show better semen parameters and hormone values than sedentary men. Eur. J. Appl. Physiol..

[6] Zhao X., Bian Y., Sun Y., Li L., Wang L., Zhao C., Shen Y., Song Q., Qu Y., Niu S. (2013). Effects of moderate exercise over different phases on age-related physiological dysfunction in testes of SAMP8 mice. Exp. Gerontol..

[7] Chigurupati S., Son T.G., Hyun D.-H., Lathia J.D., Mughal M.R., Savell J., Li S.C., Nagaraju G.P.C., Chan S.L., Arumugam T.V. (2008). Lifelong running reduces oxidative stress and degenerative changes in the testes of mice. J. Endocrinol..

[8] Szabó R., Börzsei D., Karácsonyi Z., Gesztelyi R., Nemes K., Berkó A.M., Veszelka M., Török S., Kupai K., Varga C. (2019). Postconditioning-like effect of exercis: New paradigm in experimental menopause. Am. J. Physiol. Circ. Physiol..

[9] Börzsei D., Szabó R., Hoffmann A., Harmath A., Sebestyén J., Osman J., Juhász B., Priksz D., Varga C., Pósa A. (2021). Multiple Applications of Different Exercise Modalities with Rodents. Oxidative Med. Cell. Longev..

[10] Manna I., Jana K., Samanta P.K. (2003). Effect of intensive exercise-induced testicular gametogenic and steroidogenic disorders in mature male Wistar strain rats: A correlative approach to oxidative stress. Acta Physiol. Scand..

[11] Zhou R., Ma P., Xiong A., Xu Y., Wang Y., Xu Q. (2017). Protective effects of low-dose rosuvastatin on isoproterenol-induced chronic heart failure in rats by regulation of DDAH-ADMA-NO pathway. Cardiovasc. Ther..

[12] Jimenez S.K., Jassal D.S., Kardami E., Cattini P.A. (2011). A single bout of exercise promotes sustained left ventricular function improvement after isoproterenol-induced injury in mice. J. Physiol. Sci..

[13] Díaz-Muñoz M., Alvarez-Pérez M.A., Yáñez L., Vidrio S., Martínez L., Rosas G., Yáñez M., Ramírez S., de Sánchez V.C. (2006). Correlation between oxidative stress and alteration of intracellular calcium handling in isoproterenol-induced myocardial infarction. Mol. Cell. Biochem..

[14] Varga C., Veszelka M., Kupai K., Börzsei D., Deim Z., Szabó R., Török S., Priksz D., Gesztelyi R., Juhász B. (2018). The Effects of Exercise Training and High Triglyceride Diet in an Estrogen Depleted Rat Model: The Role of the Heme Oxygenase System and Inflammatory Processes in Cardiovascular Risk. J. Sports Sci. Med..

[15] Cheng Y.S., Dai D.Z., Dai Y. (2010). Testis dysfunction by isoproterenol is mediated by upregulating endothelin receptor A, leptin and protein kinase Cvarepsilon and is attenuated by an endothelin receptor antagonist CPU0213. Reprod. Toxicol..

[16] Kumar T.R., Doreswamy K., Shrilatha B. (2002). Muralidhara Oxidative stress associated DNA damage in testis of mice: Induction of abnormal sperms and effects on fertility. Mutat. Res. Toxicol. Environ. Mutagen..

[17] Karna K.K., Soni K.K., You J.H., Choi N.Y., Kim H.K., Kim C.Y., Lee S.W., Shin Y.S., Park J.K. (2020). MOTILIPERM Ameliorates Immobilization Stress-Induced Testicular Dysfunction via Inhibition of Oxidative Stress and Modulation of the Nrf2/HO-1 Pathway in SD Rats. Int. J. Mol. Sci..

[18] Turner T.T., Lysiak J.J. (2008). Oxidative Stress: A Common Factor in Testicular Dysfunction. J. Androl..

[19] Agarwal A., Makker K., Sharma R. (2007). REVIEW ARTICLE: Clinical Relevance of Oxidative Stress in Male Factor Infertility: An Update. Am. J. Reprod. Immunol..

[20] Cocuzza M., Sikka S.C., Athayde K.S., Agarwal A. (2007). Clinical relevance of oxidative stress and sperm chromatin damage in male infertility: An evidence based analysis. Int. Braz. J. Urol..

[21] Wagner H., Cheng J.W., Ko E.Y. (2018). Role of reactive oxygen species in male infertility: An updated review of literature. Arab J. Urol..

[22] de Barboza G.D., Guizzardi S., Moine L., de Talamoni N.T. (2017). Oxidative stress, antioxidants and intestinal calcium absorption. World J. Gastroenterol..

[23] Ndrepepa G. (2019). Myeloperoxidase–A bridge linking inflammation and oxidative stress with cardiovascular disease. Clin. Chim. Acta.

[24] Aratani Y. (2018). Myeloperoxidase: Its role for host defense, inflammation, and neutrophil function. Arch. Biochem. Biophys..

[25] Samanta P.K., Manna I., Jana K. (2006). Effect of L-ascorbic acid supplementation on testicular oxidative stress and endocrine disorders in mature male rats exposed to intensive swimming exercise. Reprod. Med. Biol..

[26] Szabó R., Karácsonyi Z., Börzsei D., Juhasz B., Al-Awar A., Torok S., Berkó A.M., Takacs I., Kupai K., Varga C. (2018). Role of Exercise-Induced Cardiac Remodeling in Ovariectomized Female Rats. Oxidative Med. Cell. Longev..

[27] Yi X., Tang D., Cao S., Li T., Gao H., Ma T., Yao T., Li J., Chang B. (2020). Effect of Different Exercise Loads on Testicular Oxidative Stress and Reproductive Function in Obese Male Mice. Oxidative Med. Cell. Longev..

[28] Lin T., Wang D., Nagpal M.L., Chang W. (1994). Recombinant murine tumor necrosis factor-alpha inhibits cholesterol side-chain cleavage cytochrome P450 and insulin-like growth factor-I gene expression in rat Leydig cells. Mol. Cell. Endocrinol..

[29] Morgado J.M., Rama L., Silva I., de Jesus Inácio M., Henriques A., Laranjeira P., Pedreiro S., Rosado F., Alves F., Gleeson M. (2012). Cytokine production by monocytes, neutrophils, and dendritic cells is hampered by long-term intensive training in elite swimmers. Eur. J. Appl. Physiol..

[30] Lettieri G., D’Agostino G., Mele E., Cardito C., Esposito R., Cimmino A., Giarra A., Trifuoggi M., Raimondo S., Notari T. (2020). Discovery of the Involvement in DNA Oxidative Damage of Human Sperm Nuclear Basic Proteins of Healthy Young Men Living in Polluted Areas. Int. J. Mol. Sci..

[31] Lettieri G., Marra F., Moriello C., Prisco M., Notari T., Trifuoggi M., Giarra A., Bosco L., Montano L., Piscopo M. (2020). Molecular Alterations in Spermatozoa of a Family Case Living in the Land of Fires. A First Look at Possible Transgenerational Effects of Pollutants. Int. J. Mol. Sci..

